# Inadequate Content of Docosahexaenoic Acid (DHA) of Donor Human Milk for Feeding Preterm Infants: A Comparison with Mother’s Own Milk at Different Stages of Lactation

**DOI:** 10.3390/nu13041300

**Published:** 2021-04-15

**Authors:** Félix Castillo, Félix-Joel Castillo-Ferrer, Begoña Cordobilla, Joan Carles Domingo

**Affiliations:** 1 Service of Neonatology, Hospital Universitari Vall d’Hebron, Universitat Autònoma de Barcelona, Passeig de la Vall d’Hebron 119, E-08035 Barcelona, Spain; fecastill@vhebron.net; 2 Department of Biochemistry and Molecular Biomedicine, Faculty of Biology, University of Barcelona, Avinguda Diagonal 643, E-08028 Barcelona, Spain; bgcordobilla07@ub.edu

**Keywords:** donor human milk, preterm infants, docosahexaenoic acid

## Abstract

A cross-sectional single-center study was designed to compare the fatty acids profile, particularly docosahexaenoic acid (DHA) levels, between milk banking samples of donor human milk and mother’s own milk (MOM) for feeding preterm infants born before 32 weeks’ gestation. MOM samples from 118 mothers included colostrum (1–7 days after delivery), transitional milk (9–14 days), and mature milk (15–28 days and ≥29 days). In the n-3 polyunsaturated fatty acids (PUFAs) group, the levels of α-linolenic acid (C18:3 n3) and DHA (C22:6 n3) showed opposite trends, whereas α-linolenic acid was higher in donor human milk as compared with MOM, with increasing levels as stages of lactation progressed, DHA levels were significantly lower in donor human milk than in MOM samples, which, in turn, showed decreasing levels along stages of lactation. DHA levels in donor human milk were 53% lower than in colostrum. Therefore, in preterm infants born before 32 weeks’ gestation, the use of pasteurized donor human milk as exclusive feeding or combined with breastfeeding provides an inadequate supply of DHA. Nursing mothers should increase DHA intake through fish consumption or nutritional supplements with high-dose DHA while breastfeeding. Milk banking fortified with DHA would guarantee adequate DHA levels in donor human milk.

## 1. Introduction

Polyunsaturated fatty acids (PUFAs), omega-3 (α-linolenic acid [ALA], eicosapentaenoic acid [EPA] and docosahexaenoic [DHA] acid) and omega-6 (arachidonic acid [AA] and linoleic acid [LA]) are essential nutrients for growth, development and function [1]. DHA is derived from ALA through the enzymatic biosynthesis; ALA along with its omega-6 counterpart LA must be obtained from the diet because the human body is unable to synthesize them. PUFAs have shown to exert pleiotropic effects on cell membranes and to play a crucial role for the regulation of membrane biophysical properties, particularly as lipid mediator precursors and membrane components especially n-3 PUFAs [2,3]. DHA has demonstrated consistent beneficial healthy effects, including anti-inflammatory, antiproliferative, antiangiogenic, and antioxidant properties [4,5,6], as well as a critical involvement on underlying mechanisms of fetal neurodevelopment and immune function of newborns [7,8].

During the third trimester of pregnancy there is a selective transplacental transference of PUFAs, particularly AA and DHA, with the subsequent increase in the fetal circulation to satisfy the maximum demands of rapid development and growth of fetal brain, retina, and other tissues [9]. Cell differentiation and development of active synapses in the brain need specific requirements of DHA and AA [10]. However, premature infants are deficient in DHA for several reasons, including loss of fetal accretion of DHA that typically occurs during the third trimester and immature enzymatic systems of chain elongation and desaturation of ALA to form DHA [11,12]. These premature infants are reliant on enteral sources of DHA, most commonly through breast milk [13]. However, the DHA content in breast milk varies in direct correlation with maternal DHA intake [14,15,16], and mothers consuming a Western diet have lower levels of DHA in their milk as commonly DHA intake is below the recommended minimal intake of 450 mg/day for pregnant and lactating women [17,18]. 

Mother’s own milk (MOM) of premature infants, especially colostrum, has a high content of PUFAs, and thus DHA and AA, as compared with MOM of infants at term, and this represents a clear advantage for breastfeeding of premature infants if the mother maintains an optimal diet [19]. However, the use of donor human milk is a commonly routine practice in preterm neonates admitted to the neonatal intensive care unit (NICU) or when mothers have difficulty producing enough breast milk [20]. During the first 10 days of life of the preterm neonate, since the necessary milk volume for growth cannot be provided, parenteral nutrition is also delivered. Along the following days, the enteral feeding volume is increased, with a parallel reduction of parenteral nutrition until discontinuation when the volume of milk administered through the enteral route is sufficient for the infant nutrition.

Although feeding neonates with donor human milk from similar gestation and lactation stages would be the best option, standard donor milk banks usually provide pooled milk from lactating mothers that is far from having the composition of the MOM of preterm or very preterm infants [21,22,23]. It has been shown that DHA and AA content of human milk in mothers with preterm neonates is higher than in mothers with term neonates [24,25] and distinct patterns of changes in fatty acids levels were apparent over the course of lactation [26]. In preterm neonates, the use of parenteral nutrition and mature milk from milk banks during the first weeks of life are critical factors for increasing the risk of DHA deficiency.

Interestingly, there is a paucity of comparative data of the fatty composition of donor human milk and MOM of preterm infants. Therefore, this study was designed to assess differences in the complete fatty acids profile between donor human milk and MOM samples from mothers of preterm neonates at different stages of lactation. Assessment of differences in DHA content was the main objective of the study, given the crucial role played by this fatty acid for optimal development and function of brain neurons and retinal photoreceptors. 

## 2. Materials and Methods

### 2.1. Study Design

Between January and December 2018, a cross-sectional study was conducted at the Service of Neonatology of Hospital Universitari Vall d’Hebron in Barcelona, Spain. The primary objective of the study was to determine whether there were differences in DHA levels between donor human milk and MOM samples taken at different stages of lactation from mothers giving birth of preterm neonates born at less than 32 weeks’ gestation. The secondary objective was to assess differences in the fatty acid profile, including the distribution of saturated fatty acids (SFAs), monounsaturated fatty acids (MUFAs), n-6 PUFAs and n-3 PUFAs levels between donor human milk and MOM samples.

The study was approved by the Clinical Research Ethics Committee of our institution (code PR(AMI)287/2017, approval date 11 August 2017). Written informed consent was obtained from all participants.

### 2.2. Participants and Samples

Participants were breastfeeding women of any race or parity who delivered preterm neonates born alive before 32 weeks of pregnancy, requiring admission to the Service of Neonatology of the hospital for neonatal care. Since this was not a longitudinal study, mothers of preterm neonates in whom milk samples were collected were not the same. Data were collected prospectively from a collection of cross-sectional MOM samples at different stages of lactation, including colostrum at days 1–7 after delivery, transitional milk at days 8–14, and mature milk at 15–28 days and ≥29 days. A standardized collection protocol was used. Briefly, collection time-points were made flexible to accommodate mother requirements, so that a 24–48 h window before the extract collection was allowed. Mothers were asked to collect the sample of any breast between 10 am and 12 pm, 2–3 h after they had emptied the same breast during the last expression or breastfeed. During collection, mothers were asked to completely empty the breast with the use of an electrical breast-pump into disposable sterile bottles. From the total expressed volume, at least 1 mL were taken with a sterile enteral syringe and frozen in microtubes at −80 °C until analysis.

Human donor milk samples were obtained from the Blood and Tissue Bank, which is the entity of the Catalan Department of Health whose mission is to guarantee the supply and proper use of human blood and tissues (including human donor milk) in Catalonia. The characteristics of human donor milk were not available. According to current guidelines [27], donor human milk was pasteurized by the Holder method with maintenance of the temperature above 62.5 °C for 30 min.

### 2.3. Biochemical Analysis

Both in donor human milk and MOM samples, the panel of fatty acids analyzed included saturated fatty acids (SFAs), monounsaturated fatty acids (MUFAs), n-6 PUFAs and n-3 PUFAs. The group of SFAs included the lauric acid (C12:0), myristic acid (C14:0), palmitic acid (C16:0), stearic acid (C18:0), arachidic acid (C20:0), behenic acid (C22:0) and lignoceric acid (C24:0). The group of MUFAs included myristoleic acid (C14:1 n5), palmitoleic acid (C16:1 n7), oleic acid (C18:1 n9), cis-vaccenic acid (C18:1 n7), gondoic acid (C20:1 n9) and nervonic acid (C24:1 n9). The group of n-6 PUFAs included the linoleic acid (LA, C18:2 n6), γ-linoleic acid (C18:3 n6), cis-11,14 eicosadienoic acid (C20:2 n6), dihomo-γ-linolenic acid (C20:3 n6), AA (C20:4 n6), cis-13,16-docosadienoic acid (C22:2 n6), adrenic acid (C22:4 n6), and osbond acid or docosapentaenoic acid (DPA n6) (C22:5 n6). Finally, the group of n-3 PUFAs was composed by α-linolenic acid (ALA, C18:3 n3), (all-Z)-11,14,17-eicosatrienoic acid (C20:3 n3), (all-Z)-8,11,14,17-eicosatetraenoic acid (C20:4 n3), EPA (C20:5 n3), clupanodonic acid or docosapentaenoic acid n3 (DPA n3) (C22:5 n3) and DHA (C22:6 n3).

The determination of the fatty acid composition of milk samples was assessed by gas chromatography coupled with mass spectrometry (GC-MS). The samples were treated by an esterification method developed by Lepage and Roy [28]. The fatty acid methyl esters (FAMEs) obtained were then injected in the Shimadzu GCMS-QP2010 Plus with autosampler (Shimadzu, Kyoto, Japan). A high polarity capillary column, 15 m × 0.10 mm internal diameter, 0.10 μm film thickness, SupraWAX-280 (Teknokroma, Barcelona, Spain) was used for separation of FAMEs. The column flow rate remained constant using helium as the carrier gas, and temperature program operation was the next 130 °C with a 0.25 min hold, ramp 35 °C/min to 200 °C, 8 °C/min at 225 °C with a 3.2 min hold, and then 80 °C/min to 245 °C with a 2.75 min hold. The interface and ion source temperatures were 255 °C and 200 °C, respectively. Data analysis was performed GC-MS solution workstation software. The FAMEs were identified through electron ionization (EI) mass spectra using NIST11 library and through GC retention times, comparing with a reference FAME mixture (GLC-744, Nu-Che Prep. Inc., Elysian, MN, USA). The results were expressed in relative amounts (percentage molar of total fatty acids) of duplicate sampling.

### 2.4. Statistical Analysis

Categorical data are expressed as frequencies and percentages, and continuous data as mean and standard deviation (SD). The Shapiro-Wilk test was used to assess normal distribution of variables. Differences between the study groups (donor human milk vs. MOM) were analyzed with one-way analysis of variance (ANOVA), with Bonferroni’s correction to assess differences according to lactation stages. Statistical significance was set at *p* < 0.05. The the GraphPad Prism program, version 9.00 for Windows (GraphPad Software, San Diego, CA, USA, www.graphpad.com, accessed on 22 November 2020) was used for the analysis of data.

## 3. Results

The study sample included a convenience sample of 118 mothers of preterm neonates born at less than 32 weeks’ gestation who were admitted to the Service of Neonatology during the study period and provided written consent for breast milk sample collection. Temporary supplementation of breastfeeding with pasteurized donor human milk was required. The distribution of mothers according to provision of MOM at different stages of lactation is shown in Table 1. Statistically significant differences in maternal age, weeks of gestation, and birth weights of neonates among mothers in the different lactation stages were not found. A total of 10 samples from donor human milk were analyzed.

### 3.1. Lipid Profile of the Main Families of Fatty Acids

Donor human milk showed significantly higher relative levels of SFAs and lower relative levels of MUFAs as compared with MOM from all stages of lactation, although the composition of PUFAs and n-6 and n-3 fatty acids showed a non-significant decreasing content (Table 2). 

### 3.2. Lipid Profile of SFAs

SFAs are mostly composed by palmitic (C16:0) and stearic (C18:0) acids. The relative levels of palmitic acid were similar in donor human milk and in MOM samples from all stages of lactation, whereas relative levels of stearic acid were consistently higher in donor human milk as compared to MOM samples and differences were statistically significant from all stages of lactation (Table 2). Long-chain SFAs (C22:0 and C24:0) were similar in donor human milk and MOM samples, whereas relative levels of short-chain SFAs (C12:0 and C14:0) were lower in donor human milk and colostrum, significantly higher in transitional MOM and then lower in mature milk. 

### 3.3. Lipid Profile of MUFAs

In relation to MUFAs, oleic acid (C18:1 n9) was the main component in all study samples, followed by palmitoleic acid (C16:1 n7), cis-vaccenic acid (C18:1 n7) and gondoic acid (C20:1 n9) in minor amounts. As shown in Table 2, donor human milk showed a significantly (*p* = 0.01) lower level of oleic acid as compared with MOM from all stages of lactation. Also, oleic acid showed significantly lower levels in samples from progressive stages of lactation. Differences in the remaining MUFAs (C14:1 n5, C16:1 n7, C18:1 n7, C20:1 n9, and C24:1 n9) between donor milk and MOM samples at different stages of lactation were not statistically significant.

### 3.4. Lipid Profile of n-6 PUFAs

In relation to n-6 PUFAs, linoleic acid (C18:2 n6) precursor of fatty acids of the n-6 family, was the main component in all study samples and did not show a significant variation neither with MOM maturation and donor human milk. Long-chain PUFAs (C20:2 n6, C20:3 n6, C20:4 n6, C22:2 n6, C22:4 n6, and C22:5 n6) showed decreasing levels during the process of MOM maturation, with significant differences as compared to colostrum and donor human milk, particularly in the case of AA (C20:4 n6), C20:2 n6 and C22:4 n6 (Table 2).

### 3.5. Lipid Profile of n-3 PUFAs

**Figure 1:**
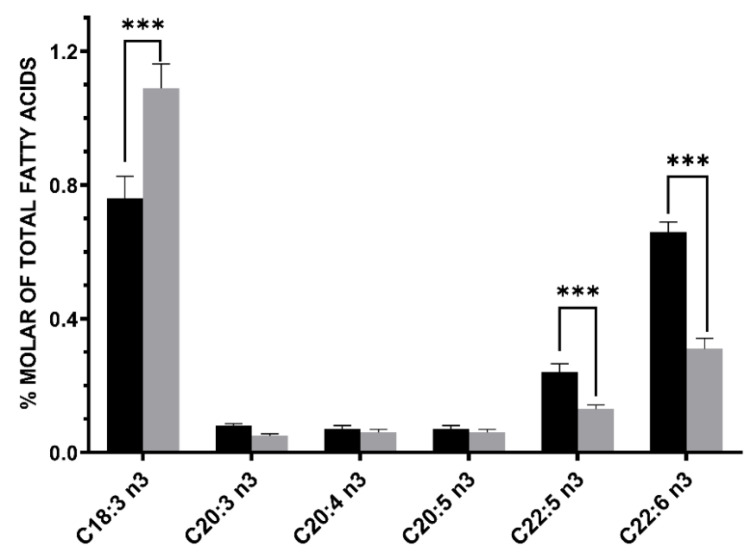
Differences in n-3 PUFAs between donor human milk (grey bar) and colostrum of mothers of preterm infants of <32 weeks’ gestation) (black bar). Donor human milk showed higher levels of α-linolenic acid (C18:3 n3, +43.4%) and about −53.0% lower levels of docosahexaenoic (DHA) acid (C22:6 n3). Asterisks indicate significant differences (*** *p* < 0.001) between the different groups.

The main components of this group of fatty acids were α-linolenic acid (C18:3 n3), DHA (C22:6 n3) and DPA n3 (C22:5 n3). The levels of α-linolenic acid and DHA showed opposite trends, whereas α-linolenic acid was higher in donor human milk as compared with MOM, with increasing levels from increasing stages of lactation, DHA levels were significantly lower in donor human milk than in MOM samples, which in turn showed decreasing levels along stages of lactation (Table 2). DPA n3 levels were significantly lower in donor human milk than in colostrum, also showing decreasing trends from colostrum to more mature stages. DHA levels in donor human milk (mean 0.31% [SD 0.04]) were 53.0% lower than in colostrum (mean 0.66 [SD 0.14]) (Figure 1).

### 3.6. Lipid Quality Indexs

**Figure 2:**
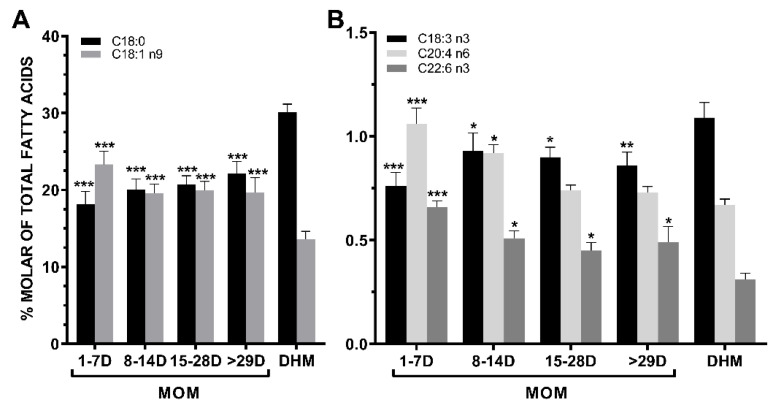
(**A**) The stearic acid (C18:0) and the oleic acid (C18:1 n-9) showed quite symmetrical but opposite trends in mothers’ own milk (MOM) during the different stages of lactation. Donor human milk (DHM) showed a significantly higher content of stearic acid and lower content of oleic acid (colostrum 1–7 days, transitional milk 8–14 days, mature mild 15–28 days and ≥29 days). (**B**) In MOM samples, α-linolenic acid (C18:3 n3) and DHA (C22:6 n3) showed a different and opposite pattern increasing and decreasing from colostrum to transitional milk, respectively. Arachidonic acid (AA) (C20:4 n6) levels in MOM samples decreased from colostrum to mature milk. Donor human milk is markedly deficient in DHA content. Asterisks indicate significant differences (* *p* < 0.05; ** *p* < 0.01; *** *p* < 0.001) in each group vs. DHM sampling points.

**Figure 3:**
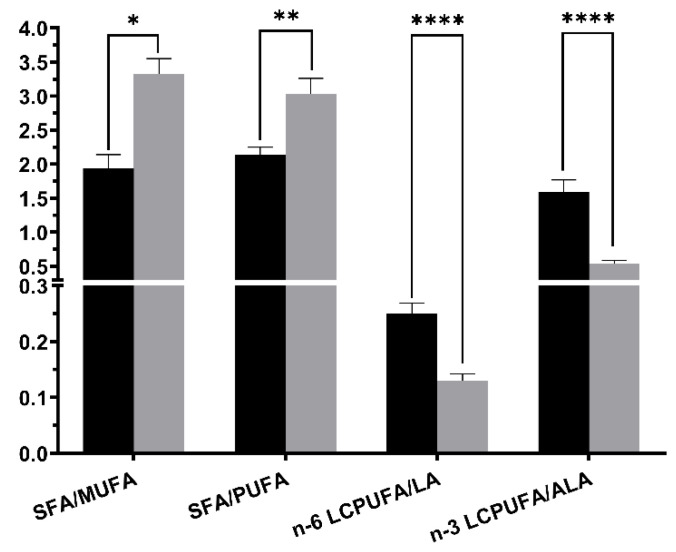
Main fatty acid ratios changes between colostrum (black bar) and human donor milk (grey bar). Asterisks indicate significant differences (* *p* < 0.05; ** *p* < 0.01; **** *p* < 0.0001) in each group among sampling points (SFA: saturated fatty acids; MUFA: monounsaturated fatty acids; PUFA: polyunsaturated fatty acids; n-6 LPUFA: n-6 long chain polyunsaturated fatty acids; LA: linoleic acid; n-3 LCPUFA: n-3 long chain polyunsaturated fatty acids; ALA: α-linoleic acid).

The most significant changes in the fatty acids profile of MOM occurring from colostrum to mature milk in mothers of premature neonates as compared with the fatty acid composition of donor human milk are shown in Figure 2. These observed changes in fatty acid composition induce that donor human milk exhibited significantly higher SFAs/MUFAs (+71.6%, *p* < 0.0001) and SFAs/PUFAs (+41.6%, *p* = 0.0056) ratios than colostrum (Figure 3). Also, in donor human milk there were significant lower levels in the relative proportion of the total n-6 and n-3 long chain-PUFAs to their precursor essential fatty acids, LA (C18:2 n6, −48.0%, *p* = 0.0005) and ALA (C18:3 n3, −66.0%, *p* = 0.0005), respectively, compared with colostrum mother milk (Figure 3).

## 4. Discussion

This study shows that donor human milk as compared with MOM samples of mothers who delivered preterm infants before 32 weeks of gestation had mainly an inadequate content of DHA, in particular significantly lower levels than in colostrum. This finding has important clinical implications, particularly when supplementation is indispensable because MOM is not available or there is not enough milk for full breastfeeding. It has been shown that pasteurization of breast milk does not affect DHA levels, although DHA content may vary significantly between banks. In a study of a large number of pooled donor breast milk samples from the Mother’s Milk Bank of Iowa [29], the average DHA provision from donor human milk was one tenth of the estimated needs of preterm neonates and does not meet recommended nutritional guidelines for optimal provision of n-3 PUFAs. Deficient composition of DHA in donor milk banks may be especially relevant for extremely low birth weight infants in the NICU [29]. Moreover, different studies have shown an association between erythrocyte phospholipid DHA content and the concentration of DHA in enriched preterm infant formulas with n-3 PUFAs [30,31]. Although different factors affect the fatty acid content of breast milk, DHA supplementation of breastfeeding mothers increases the plasma and breast milk concentrations resulting in higher infant plasma DHA concentrations [32].

An interesting and novel aspect of the study was the comparison of the fatty acid profiles of MOM samples taken at different stages of lactation in women with preterm infants. Colostrum showed a higher content of DHA, DPA n3 and AA than mature milk. In this respect, MOM at early stages of lactation is more adapted to the needs of preterm infants including higher levels of n-3 PUFAS and other nutritional contents and bioactive molecules compared to women delivering at term [19]. There is solid evidence of the important role played by DHA in the human body as essential n-3 PUFA for maintaining the structure and function of the brain and eye. In this respect, fetal development and infancy are key windows during which sufficient DHA levels are necessary for optimal cognition and visual development and performance in later life [33,34].

The higher content of α-linolenic acid in donor human milk found in our study as compared with DHA may be possibly explained by the profile of milk donors, mostly represented by mothers who had delivered infants at term [19]. This finding is in accordance to previous studies showing higher LC-PUFA proportion in preterm milk compared to term human milk [35,36]. In term infants, DHA can be synthesized from α-linolenic acid, which is in contrast to the immature enzyme systems of preterm neonates in whom DHA cannot be synthesized de novo in sufficient amounts through this enzymatic pathway and negligible stocks of them exist in their adipose tissue [37]. 

In this sense, the data in the present study show that donor human milk exhibit a substantial n-6 and n-3 LC-PUFA depletion with respect their precursors linoleic and α-linolenic acids, respectively, compared with preterm milk colostrum. These findings occur concomitantly with a raised proportion of stearic acid (C18:0) and a reduced proportion of oleic acid (C18:1), thereby increasing SFAs/MUFAs and SFAs/PUFAs ratios in donor human milk as compared with colostrum. The donor milk PUFAs/SFAs ratio was lower (0.33) than recommended value (0.45) [38]. Since, the fatty acid profile of dairy fat is important for the nutritional quality of dairy products, colostrum fat from preterm mothers had more appropriate value of lipid quality index and is a healthier option for preterm human nutrition than donor human milk. 

These findings may demonstrate the different response of the organism in view of a risk factor, increasing the quality of colostrum fat to cover specific needs of preterm infants and it is extremely important to provide adequate AA and DHA intake in the diet from early infancy. Moreover, as both types of LC-PUFA are precursor of various biologically active long-chain fatty acids derivatives, the availability of n-6 and n-3 LC-PUFA in the cells determines the ratio of the active metabolites generated.

Donor human milk is the ideal supplement for preterm neonates when MOM is insufficient. However, the administration of a higher amount of donor human milk during the first weeks of life in a preterm infant at the expense of MOM or colostrum would result in an inadequate content of DHA with the risk of not covering the optimal needs for infant growth and neurodevelopment. A systematic review and meta-analysis of 44 studies demonstrated that human milk versus preterm formula provided a clear protective effect against necrotizing enterocolitis (NEC) (with an approximate 4% reduction in incidence), and possible reduction in late-onset sepsis (LOS), severe retinopathy of prematurity (ROP), and severe NEC [39]. Low levels of DHA in preterm neonates of less than 32 weeks’ gestation have been associated with an increased risk of bronchopulmonary dysplasia (BPD) and nosocomial sepsis [40], although DHA supplementation does not appear to reduce the risk of BPD [41].

Finally, the present results should be interpreted taking into account that this was not a longitudinal study and that the number of mothers providing breast milk samples for the different stages of lactation was small. Results are presented as %mol of total fatty acids rather than through the absolute concentration as different studies have consistently shown that total fat content is significantly lower in human donor milk as compared to preterm breast milk, with both donor milk and preterm breast milk levels significantly lower than mature breast milk [42,43]. Therefore, the use of %mol of total fatty acids is also adequate for the expression of results.

## 5. Conclusions

Preterm infants born before 32 weeks’ gestation are at high risk for developing DHA deficiency, which may negatively affect growth and neurodevelopment. DHA levels of pasteurized donor milk used as a complement of MOM are insufficient to cover the needs of these premature infants. This fact is even more critical during the first two weeks of life when enteral feeding is combined with parenteral feeding. In order to achieve the recommended minimal intake of 450 mg/day of DHA for pregnant and lactating women as well as to ensure adequate DHA levels for optimal growth and neurodevelopment, especially in more immature neonates, nursing mothers of preterm infants should be recommended to increase DHA intake through fish consumption or nutritional supplements with high-dose DHA while breastfeeding. DHA supplementation of preterm infants through the enteral route may be also advisable for an adequate DHA status during development. Milk banking should also guarantee adequate DHA levels of donor human milk.

## Figures and Tables

**Table 1:** Characteristics of mothers and neonates included in the study.

Stages of Lact Ation (Number of Mothers)	Mean (Standard Deviation)
Colostrum (1–7 days) (*n* = 22)	
Maternal age, years	31.4 (5.9)
Weeks of gestation	28.5 (2.2)
Birth weight of neonates, g	1063 (382.3)
Transitional milk (8–14 days) (*n* = 28)	
Maternal age, years	31.1 (6.9)
Weeks of gestation	28.9 (2.2)
Birth weight of neonates, g	1103 (329.2)
Mature milk (15–28 days) (*n* = 50)	
Maternal age, years	33.1 (6.0)
Weeks of gestation	29.1 (2.1)
Birth weight of neonates, g	1118.6 (277.8)
Mature milk (≥29 days) (*n* = 18)	
Maternal age, years	34.5 (7.0)
Weeks of gestation	28.7 (2.3)
Birth weight of neonates, g	1123.0 (231.3)

**Table 2:** Lipid profile (% molar of total fatty acids) in donor human milk and mothers’ own milk samples from different stages of lactation.

Fatty Acids	Donor Human Milk (*n* = 10)	Stages of Lactation			
		Colostrum (1–7 Days) (*n* = 22)	Transitional (8–14 Days) (*n* = 28)	Mature (15–28 Days) (*n* = 50)	Mature (≥29 Days) (*n* = 18)
Fatty acids families					
SFAs	60.57 (4.62)	48.06 (8.18)	54.27 (7.24)	53.26 (8.65)	53.01 (8.04)
MUFAs	18.79 (3.02)	28.95 (8.47)	24.47 (5.90)	25.16 (8.80)	26.71 (8.03)
PUFAs	20.64 (3.11)	23.26 (3.21)	22.24 (2.59)	21.81 (3.22)	22.59 (5.17)
n-6 PUFAs	18.94 (3.19)	21.52 (3.0)	20.46 (2.57)	20.04 (3.22)	21.19 (3.86)
n-3 PUFAS	1.70 (0.44)	1.95 (0.80)	1.85 (0.67)	1.78 (0.42)	1.91 (0.69)
SFAs					
Lauric acid (C12:0)	4.91 (0.93)	3.82 (1.19)	6.80 (1.48)	6.54 (1.52)	6.18 (1.05)
Myristic acid (C14:0)	5.62 (1.24)	5.62 (1.26)	7.86 (2.13)	7.23 (2.05)	6.71 (1.75)
Palmitic acid (C16:0)	19.32 (1.20)	19.69 (1.96)	18.69 (1.74)	18.40 (2.33)	19.08 (2.97)
Stearic acid (C18:0)	30.11 (3.35)	18.16 (7.84)	20.08 (7.12)	20.70 (8.23)	22.16 (6.50)
MUFAs					
Oleic acid (C18:1 n9)	13.60 (3.27)	23.32 (8.11)	19.55 (6.53)	19.99 (7.94)	19.65 (8.29)
n-6 PUFAs					
LA (C18:2 n6)	16.74 (3.27)	17.17 (2.63)	17.54 (2.36)	17.07 (2.70)	18.17 (3.95)
AA (C20:4 n6)	0.67 (0.09)	1.06 (0.36)	0.92 (0.21)	0.74 (0.18)	0.73 (0.12)
n-3 PUFAs					
ALA (C18:3 n3)	1.09 (0.23)	0.76 (0.31)	0.93 (0.46)	0.90 (0.34)	0.89 (0.27)
DHA (C22:6 n3)	0.31 (0.16)	0.66 (0.14)	0.51 (0.19)	0.50 (0.26)	0.49 (0.32)
DPA (C22:5 n3)	0.13 (0.04)	0.24 (0.12)	0.15 (0.05)	0.14 (0.06)	0.15 (0.08)

SFA: saturated fatty acids; MUFAs: monounsaturated fatty acids; PUFAs: polyunsaturated fatty acids; LA: linoleic acid; AA: arachidonic acid; ALA: α-linoleic acid; DHA: docosahexaenoic acid; DPA: docosapentaenoic acid. There were statistically significant differences (*p* = 0.01) for the following comparisons: (a) SFAs, MUFAs, stearic acid, oleic acid, ALA, and DHA levels between donor human milk vs. colostrum, transitional milk, and both stages of mature milks; (b) SFAs, oleic acid, lauric acid, myristic acid, AA, ALA, DHA, and DPA n3 levels between colostrum vs. transitional milk and both stages of mature milks; (c) AA and DPA n3 levels between donor human milk vs. colostrum.

## Data Availability

Data are available from the authors (F.C. and J.C.D.) upon request.
